# Assessment of mental well-being and psychological distress in Moroccan breast cancer patients

**DOI:** 10.1590/1980-5764-DN-2024-0145

**Published:** 2024-09-23

**Authors:** Meryam Belhaj Haddou, Hicham El Mouaddib, Zakaria Belhaj Haddou, Mouna Khouchani, Noureddine El Khoudri

**Affiliations:** 1 Hassan First University of Settat, Higher Institute of Health Sciences, Laboratory of Sciences and Health Technologies, Settat, Morocco.; 2 Higher Institute of Nursing and Health Techniques, Marrakech, Morocco.; 3 Indiana State University, Terre Haute-Bayh College of Education, United States of America.; 4 University Teaching Hospital Mohammed VI, Hematology and Oncology Center, Radiation Oncology Department, Marrakesh, Morocco

**Keywords:** Breast Neoplasms, Mastectomy, Mastectomy, Segmental, Psychological Distress, Quality of Life, Psychological Well-Being, Neoplasias da Mama, Mastectomia, Mastectomia Segmentar, Angústia Psicológica, Qualidade de Vida, Bem-Estar Psicológico

## Abstract

The quality of life of breast cancer patients is strongly affected by physical pain, psychological distress, and uncertainty about vital prognosis.

**Objective:**

To assess breast cancer patients’ mental well-being and level of psychological distress at the Mohammed VI University Hospital in Marrakech.

**Methods:**

Cross-sectional study carried out from April to December 2023 at the Mohammed VI University Hospital of Marrakech. The data were collected via a questionnaire comprising a sociodemographic section and a section reserved for the Warwick-Edinburgh Mental Well-being Scale (WEMWBS) and the Kessler Psychological Distress Scale (K10). Data analysis was performed using SPSS software, version 25.

**Results:**

A total of 38.3% of patients experienced severe psychological distress. However, their mental well-being was high with a mean of 54.59 (±11.29). Older patients (>50 years) exhibited better mental well-being (56.46±10.39 vs. 52.99±11.81; p=0.020) and lower psychological distress (26.27±7.21 vs. 28.44±8.19; p=0.034) compared to their younger counterparts (≤50 years). As for the stage of cancer, patients with localized cancer presented a higher mental well-being score than patients with metastatic cancer (55.53±10.93 vs. 50.40±12.03; p=0.008). However, no statistically significant difference was recorded between mastectomy and lumpectomy patients regarding mental well-being or psychological distress.

**Conclusion:**

Breast cancer has not only a physical but also a psychological and emotional impact on patients. Thus, early diagnosis and referral to appropriate psychosocial services can improve patients’ mental well-being.

## RESUMO

A qualidade de vida das pacientes com câncer de mama é fortemente afetada pela dor física, angústia psicológica e incerteza sobre o prognóstico vital.

**Objetivo:**

Avaliar o bem-estar mental e o nível de angústia psicológica das pacientes com câncer de mama no Hospital Universitário Mohammed VI, em Marrakech.

**Métodos:**

Este foi um estudo transversal realizado de abril a dezembro de 2023 no Hospital Universitário Mohammed VI de Marrakech. Os dados foram coletados por meio de um questionário composto por uma seção sociodemográfica e uma seção reservada para a Escala de Bem-Estar Mental de Warwick-Edinburgh (*Warwick-Edinburgh Mental Well-being Scale* — WEMWBS) e a Escala de Angústia Psicológica de Kessler (K10). A análise dos dados foi realizada utilizando o software SPSS V25.

**Resultados:**

Um total de 38,3% das pacientes experimentou angústia psicológica grave. No entanto, o bem-estar mental das pacientes foi alto, com uma média de 54,59 (±11,29). As pacientes mais velhas (>50 anos) apresentaram melhor bem-estar mental (56,46±10,39 *vs*. 52,99±11,81; p=0,020) e menor angústia psicológica (26,27±7,21 *vs*. 28,44±8,19; p=0,034) em comparação com as mais jovens (≤50 anos). Quanto ao estágio do câncer, as pacientes com câncer localizado apresentaram uma pontuação de bem-estar mental mais alta do que as pacientes com câncer metastático (55,53±10,93 *vs*. 50,40±12,03; p=0,008). No entanto, não foi registrada diferença estatisticamente significativa entre as pacientes submetidas a mastectomia e aquelas submetidas a lumpectomia em relação ao bem-estar mental ou angústia psicológica.

**Conclusão:**

O câncer de mama tem não apenas um impacto físico, mas também psicológico e emocional nas pacientes. Assim, o diagnóstico precoce e o encaminhamento para serviços psicossociais adequados podem melhorar o bem-estar mental das pacientes.

## INTRODUCTION

Breast cancer ranks second among the most common cancers worldwide. It is diagnosed in approximately 30% of women^1^. In 2020, there were approximately 2.3 million new cases, resulting in approximately 685,000 deaths among women^2^. However, the survival rate in the most advanced countries is 85%^3^. In Morocco, this rate is estimated to be 83% among women aged 40 to 65 years with breast cancer^4^. Moreover, the incidence rate was estimated to be 56.4 per 100,000 people in 2020^5^. Breast cancer treatment depends on the type of cancer and its extent of spread. The main treatments are surgery, radiotherapy, chemotherapy, hormone therapy, and targeted therapy. Surgery involves the removal of the tumor and some surrounding healthy tissue during surgery. Mastectomy involves the complete removal of the breast, while lumpectomy involves the removal of the breast lesion and some of the surrounding healthy tissue while preserving the breast^6^.

This cancer is characterized by physical pain and uncertainty about a vital prognosis, which has a considerable impact on quality of life^7^. However, the side effects of treatment are more marked. Patients suffer from symptoms such as fatigue, chronic pain, nausea, alopecia, and altered body image. These symptoms are additional stressors, often associated with psychological distress manifested as anxiety, depression, or posttraumatic stress^8,9^. Indeed, the deprivation of a breast, as an organ emblematic of motherhood, sexuality, and aesthetics, is fundamentally apprehended as a reduction in physical attractiveness, fertility, and overall femininity^10^.

Consequently, women who have undergone surgery for breast cancer experience psychological difficulties following the operation. These difficulties are present regardless of the type of surgery performed and can persist after the operation, affecting patients’ quality of life over the long term^11^. Thus, the mastectomy has an impact on mental health since it causes depression, fear, despair, and a negative attitude toward one's own body^12,13^. Women who have breast cancer can be affected by mental health problems, such as anxiety, depression, and neurocognitive deficits^11^. In addition, cancer-related chemo-cerebral or cognitive disorders, such as memory and concentration problems, are also a concern in these patients, as they negatively affect quality of life, treatment compliance, and ability to work^12^.

In oncology, psychological distress is characterized as an adverse experience affecting cognitive, behavioral, emotional, social, spiritual, or psychological aspects, thus disrupting the ability to cope with the disease^14^. Distress in cancer patients can vary gradually, from a common mild vulnerability to a more severe and disabling manifestation, presenting itself in a variety of ways^8^. The diagnosis of cancer can lead to depression, anxiety, and adjustment problems. The frequency of psychological problems in people with cancer ranges from 29 to 47%^10^.

According to the World Health Organization (WHO), mental health is "a state of well-being in which the individual realizes his or her abilities, can cope with the normal stresses of life, can work productively and fruitfully, and can make a contribution to his or her community"^15^. Indeed, the key elements of mental health include:

- Emotional well-being, which focuses on managing emotions and maintaining a positive outlook;
- Psychological well-being, which concerns the ability to think, feel, and act according to one's values and goals; and
- Social well-being, which refers to the ability to form and maintain healthy relationships, communicate effectively, and participate actively in community life^16^.

Positive mental well-being is closely linked to the pursuit of happiness and satisfaction in life (hedonic), as well as to personal fulfillment through the development of capabilities, the pursuit of individual goals, productivity, the management of daily stress, and integration into the community (eudemonic)^17^.

When a patient is diagnosed with breast cancer, a significant imbalance emerges on the physical, psychological, social, emotional, and spiritual levels with profound repercussions^18^. Psychological support becomes a critical element of the care protocol, helping to sustain patients. More specifically, supporting them in the face of their condition's emotional and psychic repercussions can enhance their overall well-being and help them overcome stress^19^. However, barriers remain, such as lack of communication, awareness of psychological vulnerability, information about support services, and training for healthcare staff on the roles of psychosocial care^20-22^. Psychotherapy is, therefore, essential. Indeed, the results of one study showed a significant improvement in psychological distress after three months of psychotherapy^23^. Furthermore, the detection and management of mental health problems are essential for improving the quality of life and general well-being of breast cancer survivors^24^.

The present study aimed to assess the mental well-being and level of psychological distress of breast cancer patients at the Mohammed VI University Hospital in Marrakech.

## METHODS

This cross-sectional study was carried out from April to December 2023 at the Mohammed VI University Hospital of Marrakech. As a tertiary-level care facility, care extends to a heterogeneous range of patients, characterized by a diversity of oncological stages, age ranges, and clinical presentations. Patients are treated according to a specific protocol, with each therapeutic phase supported by evidence-based documentation in line with current scientific standards.

### Type and location of the study

The study target population involved patients recruited during follow-up, chemotherapy or radiotherapy visits at the Mohammed VI University Hospital of Marrakech. The inclusion criteria for participation comprised:

- Aged over 18 years;
- Confirmed diagnosis of breast cancer by histological examination for at least six months;
- All stages of the disease; and
- At least one anticancer treatment.

The exclusion criteria were as follows:

- Refusal to participate in the study;
- Inability to understand and speak the Moroccan dialect;
- Cognitive and/or auditory deficits; and
- Psychiatric history.

### Target population

Considering the number of 500 patients/year, a margin of error of 5% and a confidence interval (CI) of 95%, the minimum sample size was 218 patients. The formula used was $\text{n}=\frac{Z{\left(1-\frac{\alpha }{2}\right)}^{2}\times 2p(1-p)}{d^{2}}$ , where, n = minimum sample size, $Z\left(1-\frac{\alpha }{2}\right)$ = standard normal variable (at 5% type 1 error (p-value [p]<0.050) of 1.96, proportion of breast cancer (p=30%), absolute error or precision (d=5%).

### Data collection and instruments

The data were collected via a questionnaire completed by three trained interviewers. The first part of the questionnaire included patients’ sociodemographic characteristics (age, marital status, level of education, housing, monthly income, etc.), and clinical data (duration of illness, type of treatment, stage of illness, etc.). The second part included the Warwick-Edinburgh Mental Well-being Scale (WEMWBS) and the Kessler Psychological Distress Scale (K10).

The WEMWBS is a useful tool for measuring the mental well-being of overall cancer patients, including those with breast cancer^25^. It has been used in several studies to evaluate the effectiveness of interventions and to measure quality of life in cancer survivors^26-28^. The WEMWBS is a 14-item scale measuring mental well-being, scored on a 5-point scale ranging from "Never" (score 1) to "All the time" (score 5). The total score ranges from 14 to 70. A higher score indicates greater positive mental well-being.

The K10 is a widely used tool for screening psychological distress, such as anxiety or depression. It was translated into Arabic^29^, and used in cancer patients^23^. It consists of ten questions on emotional states, each accompanied by a five-level response scale. This measure can serve as a brief screening tool to identify levels of distress. The K10 score ranges from 10 to 50, with higher scores indicating increased likelihood of mental disorder or psychological distress. K10 scores are interpreted regarding likelihood of suffering from a mental disorder (psychological distress) as follows:

- 10–19 likely to be in good health;
- 20–24 likely to suffer from a mild disorder;
- 25–29 likely to suffer from a moderate disorder; and
- 30–50 likely to suffer from a severe disorder.

### Statistical analysis

Statistical analysis was carried out in the Statistical Package for Social Sciences (SPSS), version 25 after the data were prepared in Excel. Qualitative variables were analyzed by frequencies and percentages, while quantitative variables were summarized by means and standard deviations (±). The association between socio-economic variables and WEMWBS and K10 scales was tested using parametric (Pearson's chi-squared test) or nonparametric tests (Fisher's exact test), depending on the normality of the data. The correlation between the scales and the type of surgery was investigated using the chi-squared test (qualitative variables) and the Mann-Whitney U test (non-normal quantitative variables). Finally, the correlation between WEMWBS and K10 scores was determined by Pearson's correlation coefficient.

### Ethical considerations

The study was approved by the Faculty of Medicine and Pharmacy Ethics Committee in Marrakech on January 5, 2023, under 24/2022. The participation was voluntary, and the data collected were treated and reported confidentially. Participants were invited and received detailed information about the study after providing their consent by signing the appropriate form. Administrative authorization for data collection was obtained from the institution's management.

## RESULTS

Of the 230 patients in the series, 53.9% were aged 50 years or younger. The mean age of the study participants was 50 years, with 60.5% having undergone lumpectomy. The majority of participants were married (63.0%), had between one and three children (44.3%), and had no formal education (36.5%). In addition, a large proportion of participants were from urban areas (63.9%), lived with their families (61.3%), and had no work and no monthly income (78.3%). There was also a significant relationship between the type of surgery and age (p=0.046), marital status (p=0.003), education level (p=0.002), and monthly income (p=0.003) (Table 1).

**Table 1:** Patients’ characteristics by type of surgery (mastectomy vs. lumpectomy).

Variables	Modalities	All (n=230) n (%)	Mastectomy (n=116) n (%)	Lumpectomy (n=114) n (%)	p-value
Age (years)	≤50	124 (53.9)	55 (47.4)	69 (60.5)	**0.046** *
	>50	106 (46.1)	61 (52.6)	45 (39.5)	
Marital status	Married	145 (63.0)	69 (59.5)	76 (66.7)	**0.003** *
	Single	26 (11.3)	10 (8.6)	16 (14.0)	
	Divorced	23 (10.0)	9 (7.8)	14 (12.3)	
	Widow	36 (15.7)	28 (24.1)	8 (7.0)	
Level of education	Uneducated	84 (36.5)	44 (37.9)	40 (35.1)	**0.002** †
	Koranic level	33 (14.3)	25 (21.6)	8 (7.0)	
	Primary	55 (23.9)	29 (25.0)	26 (22.8)	
	Middle/High School	47 (20.4)	17 (14.7)	30 (26.3)	
	University	11 (4.8)	1 (0.9)	10 (8.8)	
Number of children	No children	30 (13.0)	12 (10.3)	18 (15.8)	0.065*
	1–3	102 (44.3)	46 (39.7)	56 (49.1)	
	≥4	98 (42.6)	58 (50.0)	40 (35.1)	
Habitat	Urban	147 (63.9)	72 (62.1)	75 (65.8)	0.557*
	Rural	83 (36.1)	44 (37.9)	39 (34.2)	
Live	With family	141 (61.3)	67 (57.8)	74 (64.9)	0.265*
	Alone	89 (38.7)	49 (42.2)	40 (35.1)	
Work	Yes	50 (21.7)	24 (20.7)	26 (22.8)	0.697*
	No	180 (78.3)	92 (79.3)	88 (77.2)	
Health coverage	Yes	178 (77.4)	89 (76.7)	89 (78.1)	0.638*
	No	52 (22.6)	27 (23.3)	25 (21.9)	
Monthly income	No Income	180 (78.3)	93 (80.2)	87 (76.3)	**0.033** †
	<2500 Dhs	24 (10.4)	16 (13.8)	8 (7.0)	
	2500-5000 Dhs	20 (8.7)	6 (5.2)	14 (12.3)	
	5000-7000 Dhs	6 (2.6)	1 (0.9)	5 (4.4)	

Abbreviations: Dhs, Dirham Moroccan currency.

Notes: Bold indicates p-value<0.05.

* χ^2^ test;

† Fisher's exact test.

Overall, 60.0% of patients were diagnosed more than two years ago, with 57.8% and 62.3% having undergone mastectomy and lumpectomy, respectively. Moreover, 43.9% of patients had associated pathologies. This percentage was even greater among mastectomy patients (54.3%), and among these same patients, 31.9% had advanced cancer. In addition, 94.8% underwent chemotherapy, and 50.0% received combined radiotherapy and hormone therapy. The results revealed a significant relationship between type of medical treatment and type of surgery (p<0.001), as well as between tumor stage (p=0.004) and presence of associated pathologies (p<0.001) (Table 2).

**Table 2:** Clinical characteristics of patients according to type of surgery (mastectomy vs. lumpectomy).

Variables	Modalities	All (n=230) n (%)	Mastectomy (n=116) n (%)	Lumpectomy (n=114) n (%)	p-value*
Time since diagnosis	≤2 years	92 (40.0)	49 (42.2)	43 (37.7)	0.484
	>2 years	138 (60.0)	67 (57.8)	71 (62.3)	
Associated pathologies	Yes	101 (43.9)	63 (54.3)	38 (33.3)	**0.001**
	No	129 (56.1)	53 (45.7)	76 (66.7)	
Family antecedents of cancer	Yes	113 (49.1)	58 (50.0)	55 (48.2)	0.790
	No	117 (50.9)	58 (50.0)	59 (51.8)	
Cancer stage	Metastatic	42 (18.3)	37 (31.9)	5 (4.4)	**0.004**
	Located	188 (81.7)	79 (68.1)	109 (95.6)	
Chemotherapy	Yes	175 (76.1)	110 (94.8)	65 (57.0)	**0.000**
	No	55 (23.9)	6 (5.2)	49 (43.0)	
Radiotherapy	Yes	155 (67.4)	58 (50.0)	97 (85.1)	**0.000**
	No	75 (32.6)	58 (50.)	17 (14.9)	
Hormonotherapy	Yes	94 (40.9)	58 (50.0)	36 (31.6)	**0.000**
	No	136 (59.1)	58 (50.0)	78 (68.4)	

Notes: Bold indicates p-value<0.05;

* χ^2^ test.

Table 3 shows that mental well-being in the study population was high. Lumpectomy patients had a higher mean score than mastectomy patients (55.74±11.48 vs. 53.45±11.02). In addition, 38.3% experienced severe psychological distress. There were no statistically significant differences in mental well-being or psychological distress between the two categories of patients.

**Table 3:** Comparison of WEMWBS and K10 according to type of surgery.

WEMWBS		All (n=230) Mean (SD)	Mastectomy (n=116) Mean (SD)	Lumpectomy (n=114) Mean (SD)	p-value
		54.59 (11.29)	53.45 (11.02)	55.74 (11.48)	0.077*
K10	Probability of being in good health	34 (14.8)	15 (12.9)	19 (16.7)	0.217†
	Probability of suffering from a mild disorder	46 (20.0)	20 (17.2)	26 (22.8)	
	Probability of suffering from a moderate disorder	62 (27.0)	38 (32.8)	24 (21.1)	
	Probability of suffering from a severe disorder	88 (38.3)	43 (37.1)	45 (39.5)	

Abbreviations: WEMWBS, Warwick-Edinburgh Mental Well-being Scale; K10, Kessler Psychological Distress Scale; SD, standard deviation. Notes: p<0.050.

* Mann-Whitney U test;

† χ^2^ test.

Table 4 shows that there is a strong negative correlation between mental well-being (WEMWBS) and the psychological distress scale (K10), with r=-0.478 and p<0.001. Indeed, the higher the WEMWBS score, the lower the K10 score (Table 4).

**Table 4:** Correlation between mental well-being and psychological distress.

		WEMWBS	K10
WEMWBS	Pearson correlation	1	-.478*
	Sig, (bilateral)		**<0.001** †
	number	230	230
K10	Pearson Correlation	-0.478*	1
	Sig, (bilateral)	**<0.001** †	
	number	230	230

Abbreviations: WEMWBS, Warwick-Edinburgh Mental Well-being Scale; K10, Kessler Psychological Distress Scale; Sig, significance.

Notes:

* The correlation is significant at the 0.010 (bilateral);

† The correlation is significant at the 0.050 (bilateral).

Data revealed a significant relationship between age and the WEMWBS score as well as psychological distress (p=0.020, p=0.034, respectively). Indeed, older patients (>50 years) showed better mental well-being (56.46±10.39 vs. 52.99±11.81) than younger patients. Similarly, a significant relationship was observed between the WEMWBS score and cancer stage (p=0.008). Patients with localized cancer had a higher WEMWBS score than patients with metastatic cancer (55.53±10.93 vs. 50.40±12.03) (Tables 5 and 6).

**Table 5:** Correlation between socio-economic variables and mental well-being with psychological distress in breast cancer patients (n=230).

Variables	Scales	Modalities	Mean (SD)	p-value*
Age	WEMWBS	≤50 years	52.99 (11.81)	**0.020**
		>50 years	56.46 (10.39)	
	K-10	≤50 years	28.44 (8.19)	**0.034**
		>50 years	26.27 (7.21)	
Marital status	WEMWBS	Married	54.72 (9.68)	0.785
		Not married	54.36 (13.67)	
	K-10	Married	27.64 (7.89)	0.630
		Not married	27.09 (7.72)	
Level of education	WEMWBS	Instructed	55.66 (10.65)	0.057
		Uninstructed	53.56 (11.83)	
	K-10	Instructed	26.83 (7.27)	0.269
		Uninstructed	28.03 (8.29)	
Number of children	WEMWBS	No children	53.9 (14.1)	0.522
		Has children	54.7 (10.85)	
	K-10	No children	27.27 (7.71)	0.674
		Has children	27.47 (7.85)	
Habitat	WEMWBS	Urban	54.95 (11.2)	0.520
		Rural	53.95 (11.49)	
	K-10	Urban	26.86 (7.8)	0.133
		Rural	28.47 (7.78)	
Live alone	WEMWBS	With family	54.22 (11.95)	0.531
		Alone	55.18 (10.21)	
	K-10	With family	28.18 (8.58)	0.071
		Alone	26.27 (6.28)	
Work	WEMWBS	Yes	53.26 (11.81)	0.347
		No	54.96 (11.15)	
	K-10	Yes	27.1 (7.28)	0.729
		No	27.53 (7.97)	
Monthly income	WEMWBS	No Income	54.92 (10.93)	0.114
		At monthly income	53.4 (12.55)	
	K-10	No Income	27.66 (7.94)	0.827
		At monthly income	26.64 (7.35)	
Health coverage	WEMWBS	No coverage	51.9 (10.73)	0.112
		Health coverage	55.38 (11.36)	
	K-10	No coverage	29.19 (8.04)	0.391
		Health coverage	26.93 (7.69)	

Abbreviations: WEMWBS, Warwick-Edinburgh Mental Well-being Scale; K10, Kessler Psychological Distress Scale; SD, standard deviation.

Notes: Bold indicates p-value<0.05;

* Student's t-test.

**Table 6:** Correlation between clinical variables and mental well-being with psychological distress in breast cancer patients (n=230).

Variables	Scales	Modalities	Mean (SD)	p-value*
Duration of the disease	WEMWBS	≤2years	54.05 (12.69)	0.557
		>2years	54.95 (10.29)	
	K-10	≤2years	28.07 (8.46)	0.322
		>2years	27.02 (7.36)	
Associated pathologies	WEMWBS	Yes	53.52 (12.09)	0.206
		No	55.43 (10.6)	
	K-10	Yes	28.38 (8.01)	0.108
		No	26.71 (7.6)	
Family antecedents of cancer	WEMWBS	Yes	55.77 (11.2)	0.120
		No	53.45 (11.31)	
	K-10	Yes	27.68 (8.32)	0.645
		No	27.21 (7.32)	
Chemotherapy	WEMWBS	Yes	54.75 (11.45)	0.697
		No	54.07 (10.85)	
	K-10	Yes	27.18 (7.6)	0.366
		No	28.27 (8.49)	
Radiotherapy	WEMWBS	Yes	55.28 (11.08)	0.186
		No	53.17 (11.67)	
	K-10	Yes	27.28 (7.7)	0.653
		No	27.77 (8.08)	
Hormonotherapy	WEMWBS	Yes	54.97 (11.09)	0.675
		No	54.33 (11.46)	
	K-10	Yes	27.02 (7.82)	0.502
		No	27.73 (7.82)	
Cancer stage	WEMWBS	Metastatic	50.4 (12.03)	**0.008**
		Located	55.53 (10.93)	
	K-10	Metastatic	28.62 (8.43)	0.280
		Located	27.18 (7.67)	

Abbreviations: WEMWBS, Warwick-Edinburgh Mental Well-being Scale; K10, Kessler Psychological Distress Scale; SD, standard deviation.

Notes: Bold indicates p-value<0.05;

* Student's t-test.

## DISCUSSION

Cancer is a difficult disease that affects patients and their families both physically and emotionally. Indeed, emotional distress is a critical problem for these patients, adversely of affecting their quality of life. Recent increases in cancer incidence and oncology-related problems have motivated researchers to analyze the causes and effects of emotional distress in these patients^30,31^.

The results showed a significant association between the type of surgery and comorbidities (p=0.001), and cancer stage (p=0.004). The majority of patients (60.0%) were aware of their diagnosis more than two years previously. Indeed, when disease progression exceeds 18 months, psychological distress persists at a high level in breast cancer patients^32^. Moreover, the presence of comorbidities is associated with a greater risk of psychological distress^33^. Similarly, patients with metastatic breast cancer have higher rates of potential depression and psychosocial problems than patients with early-stage breast cancer^34,35^. Chemotherapy patients may suffer from anxiety and psychological distress. Indeed, the latter is positively correlated with anxiety scores in patients undergoing chemotherapy after breast cancer surgery^36^.

Furthermore, 38.3% of patients presented with severe psychological distress (K10≥30). This percentage is almost similar (39.0%) to that in a study conducted in a Muslim context^37^, but lower (45.0%) than in a non-Muslim context^38^. Indeed, K10 scores above 30 indicate that patients are more likely to be diagnosed with an internalizing disorder^39^. However, Islamic psychotherapy can be useful in helping these patients heal their emotions, behaviors, and spirit, and in strengthening their spirituality^40^. Despite this, breast tissue loss can still have an impact on body image and self-esteem such that patients feel less feminine. As a result, mastectomy has a negative impact on psychological, emotional, and social well-being^41,42^.

The results of the present study also showed that there was no significant difference between mental well-being, psychological distress, and type of surgery. In the same vein, Kiebert et al. showed that regardless of the type of surgery performed, whether mastectomy or breast-conserving surgery, there was no change in feelings of fear or worry^43^. However, despite high psychological distress in 39.0% of patients, a surprising level of mental well-being is observed in this population. Two key factors may explain this phenomenon. First, patient resilience plays a crucial role. Studies reveal a positive correlation between resilience and reduced psychological distress^9^. This ability to overcome adversity enables patients to maintain a high level of mental well-being in the face of breast cancer. On the other hand, social support in Moroccan society is also an essential element. Moroccan culture values the accompaniment of sick people, translating into devotion and attachment to them^44^. This social support, from family, friends, and the community mitigates the negative effects of illness and promotes patients’ mental well-being.

Overall, the study population's WEMWBS score was higher than the standard mean score (54.59±11.29). Indeed, breast cancer is a frightening experience for patients, since feelings such as fear of death, anger, and confusion are considered normal throughout the treatment process. Patients who are mentally well and satisfied with their lives are able to make sense of them. Such patients have good control over events through their positive attitudes and emotions. Those with low mental well-being experience negative emotions, anxiety, and depression as well as stress^45^. At this point, religious beliefs can help patients establish balance in their lives. Indeed, the participation of patients undergoing chemotherapy in spiritual and religious activities was supportive of overcoming the disease^46^. Moreover, positive religious coping involves the constructive use of religion or spirituality to find comfort, hope, social support, or other forms of adaptation in the face of difficulties^47,48^.

Based on the results of the present study, it was confirmed that age and cancer stage are essential factors influencing mental well-being and psychological distress in breast cancer patients. Indeed, patients under 55 years of age have a low level of psychological well-being^49^, which could be explained by the stress associated with this age group. On the other hand, older patients face health problems related to aging, such as cognitive impairment. Compared with younger women, older women are at higher risk of developing cognitive decline before treatment. This significantly impacts their quality of life and their ability to cope with the stresses and challenges of the disease. Another study confirmed that young age and advanced tumor stage are predictive factors for mental disorders^50^. The stage of cancer at diagnosis can impact psychological health, with advanced stages being predictive of anxiety and distress^51^. However, another study showed that there is no significant relationship between demographic variables and the mental health of cancer patients^52^, which implies the presence of other cultural and religious factors that need to be explored in future research. Therefore, healthcare professionals should consider age and tumor stage to be predictive factors when providing emotional or psychological support^53,54^.

The present study is the first in Morocco to combine the WEMWBS and K10 scales in a population of breast cancer patients; however, it has several limitations:

- The WEMWBS was adapted into Arabic, but has not been subjected to a psychometric validation process;
- The study sample cannot be representative of all Moroccan women with breast cancer; and
- The results obtained concerning well-being and psychological distress need to be investigated over a long period.

In conclusion, breast cancer not only is a physical health problem but also has deleterious consequences for patients’ mental and emotional well-being. The stress and anxiety associated with breast cancer diagnosis and treatment can be overwhelming, leading to increased levels of depression, fear, and uncertainty. It is therefore crucial to recognize and address the psychological dimensions of breast cancer to provide comprehensive care and support to patients. To this end, screening for distress and prompt referral to appropriate psychosocial services are recommended. Psychosocial education and interventions such as physical activity (e.g. yoga, tai chi, aerobics, and meditation) have also been shown to improve quality of life and compliance. Psychotherapeutic and supportive approaches should, therefore, be offered throughout the patient's oncological history. To provide comprehensive screening and guidance to breast cancer patients, caregivers need to understand risk factors and signs of distress and anxiety.
